# Children’s erythrocyte fatty acids are associated with the risk of islet autoimmunity

**DOI:** 10.1038/s41598-021-82200-9

**Published:** 2021-02-11

**Authors:** Sari Niinistö, Iris Erlund, Hye-Seung Lee, Ulla Uusitalo, Irma Salminen, Carin Andrén Aronsson, Hemang M. Parikh, Xiang Liu, Sandra Hummel, Jorma Toppari, Jin-Xiong She, Åke Lernmark, Annette G. Ziegler, Marian Rewers, Beena Akolkar, Jeffrey P. Krischer, David Galas, Siba Das, Nikita Sakhanenko, Stephen S. Rich, William Hagopian, Jill M. Norris, Suvi M. Virtanen, the TEDDY Study Group, Aaron Barbour, Kimberly Bautista, Judith Baxter, Daniel Felipe-Morales, Kimberly Driscoll, Brigitte I. Frohnert, Marisa Stahl, Patricia Gesualdo, Michelle Hoffman, Rachel Karban, Edwin Liu, Stesha Peacock, Hanan Shorrosh, Andrea Steck, Megan Stern, Erica Villegas, Kathleen Waugh, Olli G. Simell, Annika Adamsson, Suvi Ahonen, Mari Åkerlund, Leena Hakola, Anne Hekkala, Henna Holappa, Heikki Hyöty, Anni Ikonen, Jorma Ilonen, Sinikka Jäminki, Sanna Jokipuu, Leena Karlsson, Jukka Kero, Miia Kähönen, Mikael Knip, Minna-Liisa Koivikko, Merja Koskinen, Mirva Koreasalo, Kalle Kurppa, Jarita Kytölä, Tiina Latva-Aho, Katri Lindfors, Maria Lönnrot, Elina Mäntymäki, Markus Mattila, Maija Miettinen, Katja Multasuo, Teija Mykkänen, Tiina Niininen, Mia Nyblom, Sami Oikarinen, Paula Ollikainen, Zhian Othmani, Sirpa Pohjola, Petra Rajala, Jenna Rautanen, Anne Riikonen, Eija Riski, Miia Pekkola, Minna Romo, Satu Ruohonen, Satu Simell, Maija Sjöberg, Aino Stenius, Päivi Tossavainen, Mari Vähä-Mäkilä, Sini Vainionpää, Eeva Varjonen, Riitta Veijola, Irene Viinikangas, Desmond Schatz, Diane Hopkins, Leigh Steed, Jennifer Bryant, Katherine Silvis, Michael Haller, Melissa Gardiner, Richard McIndoe, Ashok Sharma, Stephen W. Anderson, Laura Jacobsen, John Marks, Ezio Bonifacio, Cigdem Gezginci, Anja Heublein, Eva Hohoff, Annette Knopff, Charlotte Koch, Sibylle Koletzko, Claudia Ramminger, Roswith Roth, Jennifer Schmidt, Marlon Scholz, Joanna Stock, Katharina Warncke, Lorena Wendel, Christiane Winkler, Daniel Agardh, Maria Ask, Rasmus Bennet, Corrado Cilio, Susanne Dahlberg, Helene Engqvist, Emelie Ericson-Hallström, Annika Björne Fors, Lina Fransson, Thomas Gard, Monika Hansen, Hanna Jisser, Fredrik Johansen, Berglind Jonsdottir, Helena Elding Larsson, Marielle Lindström, Markus Lundgren, Marlena Maziarz, Maria Månsson-Martinez, Jessica Melin, Zeliha Mestan, Caroline Nilsson, Karin Ottosson, Kobra Rahmati, Anita Ramelius, Falastin Salami, Anette Sjöberg, Birgitta Sjöberg, Carina Törn, Åsa Wimar, Michael Killian, Claire Cowen Crouch, Jennifer Skidmore, Masumeh Chavoshi, Arlene Meyer, Jocelyn Meyer, Denise Mulenga, Nole Powell, Jared Radtke, Matei Romancik, Shreya Roy, Davey Schmitt, Sarah Zink, Dorothy Becker, Margaret Franciscus, MaryEllen Dalmagro-Elias Smith, Ashi Daftary, Mary Beth Klein, Chrystal Yates, Michael Abbondondolo, Lori Ballard, Rasheedah Brown, Stephen Dankyi, David Hadley, Wendy McLeod, Aubrie Merrell, Steven Meulemans, Ryan Quigley, Liping Yu, Dongmei Miao, Polly Bingley, Alistair Williams, Kyla Chandler, Ilana Kelland, Yassin Ben Khoud, Huma Zahid, Matthew Randell, Jouko Sundvall, Nina Kangas, Petra Arohonka, Masumeh Chavoshi, Jared Radtke, Sarah Zink, Previously Henry Erlich, Steven J. Mack, Anna Lisa Fear, Wei-Min Chen, Suna Onengut-Gumuscu, Emily Farber, Rebecca Roche Pickin, Jonathan Davis, Jordan Davis, Dan Gallo, Jessica Bonnie, Paul Campolieto, Sandra Ke, Niveen Mulholland, Kasia Bourcier, Thomas Briese, Suzanne Bennett Johnson, Eric Triplett

**Affiliations:** 1 Health and Well-Being Promotion Unit, Public Health and Welfare Department, Finnish Institute for Health and Welfare, P.O. Box 30, 00271 Helsinki, Finland; 2 Department of Government Services, Finnish Institute for Health and Welfare, Helsinki, Finland; 3 Health Informatics Institute, Morsani College of Medicine, University of South Florida, Tampa, USA; 4 Department of Clinical Sciences, Lund University, CRC, Skåne University Hospital, Malmö, Sweden; 5 Institute of Diabetes Research, Helmholtz Zentrum München and Forschergruppe Diabetes, Klinikum Rechts Der Isar, Technische Universität München and Forschergruppe Diabetes e.V., Munich, Germany; 6 Department of Physiology, University of Turku, Turku, Finland; 7 Medical College of Georgia, Augusta University, Augusta, GA USA; 8 Barbara Davis Center for Childhood Diabetes, University of Colorado School of Medicine, Aurora, USA; 9 National Institute of Diabetes and Digestive and Kidney Diseases, National Institutes of Health, Bethesda, MD USA; 10 Pacific Northwest Research Institute, Seattle, WA USA; 11 University of Virginia School of Medicine, Virginia, USA; 12 Department of Epidemiology, Colorado School of Public Health, University of Colorado Denver, Aurora, USA; 13 Faculty of Social Sciences/Health Sciences, Tampere University and Center for Child Health Research, Tampere University and Tampere University Hospital, Tampere, Finland; 14 The Science Center of Pirkanmaa Hospital District, Tampere, Finland; 15 Hospital District of Southwest Finland, Turku University Hospital, Turku, Finland; 16 University of Oulu, Oulu, Finland; 17 Oulu University Hospital, Oulu, Finland; 18 University of Kuopio, Kuopio, Finland; 19 Pediatric Endocrine Associates, Atlanta, USA; 20 Center for Regenerative Therapies, TU Dresden, Dresden, Germany; 21 Department of Nutritional Epidemiology, University of Bonn, Bonn, Germany; 22 Dr. Von Hauner Children’s Hospital, Department of Gastroenterology, Ludwig Maximillians University Munich, Munich, Germany; 23 Children’s Hospital of Pittsburgh of UPMC, Pittsburgh, USA; 24 Bristol Medical School, University of Bristol, Bristol, UK; 25 Center for Genetics, Children’s Hospital Oakland Research Institute, Oakland, USA; 26 NIDDK Biosample Repository at Fisher BioServices, Rockville, USA; 27 National Institutes of Allergy and Infectious Diseases, Palo Alto, USA; 28 Columbia University, New York, USA; 29 Florida State University, Tallahassee, USA; 30 University of Florida, Gainesville, USA

**Keywords:** Biomarkers, Endocrinology, Pathogenesis, Risk factors

## Abstract

Our aim was to investigate the associations between erythrocyte fatty acids and the risk of islet autoimmunity in children. The Environmental Determinants of Diabetes in the Young Study (TEDDY) is a longitudinal cohort study of children at high genetic risk for type 1 diabetes (n = 8676) born between 2004 and 2010 in the U.S., Finland, Sweden, and Germany. A nested case–control design comprised 398 cases with islet autoimmunity and 1178 sero-negative controls matched for clinical site, family history, and gender. Fatty acids composition was measured in erythrocytes collected at the age of 3, 6, and 12 months and then annually up to 6 years of age. Conditional logistic regression models were adjusted for HLA risk genotype, ancestry, and weight z-score. Higher eicosapentaenoic and docosapentaenoic acid (n − 3 polyunsaturated fatty acids) levels during infancy and conjugated linoleic acid after infancy were associated with a lower risk of islet autoimmunity. Furthermore, higher levels of some even-chain saturated (SFA) and monounsaturated fatty acids (MUFA) were associated with increased risk. Fatty acid status in early life may signal the risk for islet autoimmunity, especially n − 3 fatty acids may be protective, while increased levels of some SFAs and MUFAs may precede islet autoimmunity.

## Introduction

Fatty acids are important constituents of complex lipids and cell membranes, affecting their physiological properties and cellular functions. They are also well-established precursors of lipid mediators, e.g. eicosanoids, which are involved in various inflammatory reactions and affect immunity, lipid and glucose metabolism, as well as insulin responses^1–4^. Fatty acids may play a role in the development of type 1 diabetes, an autoimmune disease characterized by the destruction of pancreatic insulin-producing beta cells. The strongest evidence concerns long-chain *n* − *3* polyunsaturated fatty acid (PUFA) intake or status during infancy and childhood, which protected from islet autoimmunity^5–7^, and showed an interaction with breastfeeding^7^. However, n − 3 PUFAs were not associated with the progression from islet autoimmunity to type 1 diabetes^8^. In addition, other fatty acids than n − 3 PUFAs have been associated with islet autoimmunity. Serum breast milk-derived fatty acids during infancy were protectively associated with primary insulin autoimmunity^7^, while dairy-derived fatty acids during later childhood were directly associated with islet autoimmunity^9^. Also, metabolomic studies investigating other lipids have reported that early metabolic lipid dysregulation preceding islet autoimmunity in children who later progressed to type 1 diabetes^10–14^.

Different types of fatty acids biomarkers have been used in previous studies, most commonly from serum or erythrocytes. Erythrocyte fatty acid composition is considered the most stable biomarker, reflecting long-term dietary intake or endogenous biosynthesis and metabolism several weeks or months back^15^. Some of the individual fatty acids are more useful as dietary biomarkers than others, because they reflect changes in dietary intake better. This includes fatty acids belonging to the *n* − *6* and *n* − *3* pathways^16,17^. In line with the definition, the essential fatty acids *n* − *6* linoleic acid (LA) and *n* − *3* alpha-linolenic acid (ALA) are solely obtained from the diet. These compounds are metabolized by the *n* − *3* and *n* − *6* pathways to form longer chain fatty acids^18^, which serve as precursors for lipid mediators. Main dietary sources of LA and ALA are vegetable oils, while longer chain *n* − *3* fatty acids eicosapentaenoic (EPA), docosapentaenoic (DPA) and docosahexaenoic acid (DHA) are mainly obtained from marine foods. Other types of fatty acid biomarkers, e.g. the odd-chain fatty acids pentadecanoid acid (15:0) and heptadecanoid acid (17:0), as well as conjugated linoleic acid (CLA) (18:2n − 7), have been applied as biomarkers of dairy fat^19,20^, although it is well known that fatty acids are rarely specific for any dietary source and they are often produced endogenously also^21–23^. This is the case especially for the largest pool of fatty acids on cell membranes, the even-chain saturated fatty acids (SFA). The even-chain SFA and monounsaturated fatty acids (MUFA) are not considered good biomarkers of dietary intake, because they mostly reflect endogenous fatty acid metabolism and biosynthesis in the liver by a process that produces fatty acids from glucose mainly (de novo lipogenesis), as well as metabolism of shorter chain fatty acids^24^.

Type 1 diabetes may consist of different disease endotypes^25^. First emerging autoantibody may reflect different etiology of endotypes, and different genes and environmental exposures may be associated with them^7,26,27^. The aim of the current study was to evaluate the associations of erythrocyte fatty acid composition during infancy and childhood with the different types of islet autoimmunity. Our hypothesis was that the long-chain *n* − *3* PUFAs are associated with reduced risk.

## Results

### Erythrocyte fatty acid composition in infancy and the risk of the risk islet autoimmunity

Characteristics of children by matching factors are presented in Table 1, and erythrocyte fatty acid status in children in Supplementary information Table 1. Higher proportion of EPA and DPA at 3 months was associated with a lower risk for islet autoimmunity. In contrast, oleic acid (18:1*n* − *9*) at 3 months and palmitic acid (16:0) at 6 months were associated with an increased risk of islet autoimmunity (Table 2). Erythrocyte fatty acid composition of infants differed according to breastfeeding status defined as consumption of any breastmilk (yes/no) at the age of 3 or 6 months (Table 3). Non-breastfed infants exhibited higher levels of oleic acid (18:1*n* − *9*) and palmitic acid (16:0) than breastfed children, and these fatty acids were associated with an increased risk of islet autoimmunity. ALA (18:3*n* − *3*), LA (18:2*n* − *6*) and docosanoid acid (22:0) showed an interaction with breastfeeding at 3 months on the risk of islet autoimmunity (ALA *p* = 0.024, LA *p* = 0.038, docosanoid acid *p* = 0.027). In non-breastfed infants, ALA (OR 0.35, 95% CI 0.15–0.83) and LA (0.18, 0.04–0.76) were associated with a lower risk of islet autoimmunity, while no associations were observed in breastfed infants (ALA 1.09, 0.64–1.84; LA 1.06, 0.42–2.68). Docosanoid acid (22:0) was associated with an increased risk in non-breastfed infants (non-breastfed 3.31, 1.08–10.14; breastfed 0.82, 0.41–1.64).

**Table 1:** Characteristics of TEDDY children with islet autoimmunity and control children.

	Case children Total n = 398	Control children Total n = 1178
**Clinical center, n (%)**		
Colorado	56 (14.1)	162 (13.8)
Georgia	27 (6.8)	78 (6.6)
Washington	36 (9.1)	107 (9.1)
Finland	113 (28.4)	339 (28.8)
Germany	35 (8.8)	105 (8.9)
Sweden	131 (32.9)	387 (32.9)
**Sex, n (%)**		
Female	178 (44.7)	530 (45.0)
Male	220 (55.3)	648 (55.0)
**Status regarding first degree relative**		
First degree relative with type 1 diabetes	88 (22.1)	259 (22.0)
General population	310 (77.9)	917 (78.0)
**HLA genotype, n (%)**		
High risk (DR3/4)	210 (52.8)	420 (35.7)
Moderate risk (other genotypes)	187 (47.0)	747 (63.4)
Missing	1 (0.2)	11 (0.9)
**Ancestry, mean (SD)**		
Principal component 1	0.0017 (0.0074)	0.0013 (0.0078)
Principal component 2	− 0.0003 (0.0109)	− 0.0016 (0.0094)
**Breastfed, n (%)**		
At 3 months	307 (77.1)	903 (76.7)
At 6 months	252 (63.3)	778 (66.0)
Missing information	1 (0.3)	4 (0.3)
**Weight z score, mean (SD)**		
At 3 months	0.68 (0.95)	0.41 (1.03)
At 6 months	0.47 (1.00)	0.24 (1.01)
Over 1–6 years	0.20 (1.04)	0.01 (0.99)

**Table 2:** The risk of islet autoimmunity associated with erythrocyte fatty acid status in TEDDY nested case–control study.

Relative percentage of total fatty acids in erythrocyte membrane	Islet autoimmunity, cases n = 398					
	3 months		6 months		Mean over 1–6 years	
	OR (95% CI)^a^	*p* value	OR (95% CI)^a^	*p* value	OR (95% CI)^a^	*p* value
**SFA**						
Myristic acid 14:0	0.95 (0.52–1.74)	0.861	1.26 (0.74–2.13)	0.395	0.87 (0.42–1.81)	0.706
Pentadecanoic acid 15:0	1.19 (0.68–2.07)	0.550	0.94 (0.53–1.67)	0.821	0.54 (0.27–1.05)	0.068
Palmitic acid 16:0	2.31 (0.89–5.99)	0.085	3.35 (1.30–8.65)	0.013	3.18 (0.82–12.41)	0.096
Heptadecanoic acid 17:0	1.95 (0.83–4.60)	0.125	1.19 (0.55–2.58)	0.655	0.88 (0.31–2.50)	0.808
iso − heptadecanoic acid i17:0	0.98 (0.76–1.27)	0.882	0.87 (0.68–1.11)	0.264	0.68 (0.45–1.02)	0.061
Stearic acid 18:0	2.40 (0.81–7.16)	0.115	2.21 (0.71–6.85)	0.170	4.70 (1.48–14.89)	0.009
Eicosanoid acid 20:0	1.34 (0.76–2.37)	0.309	1.26 (0.70–2.26)	0.441	1.26 (0.59–2.68)	0.546
Docosanoic acid 22:0^b^	1.23 (0.70–2.16)	0.478	1.34 (0.72–2.50)	0.348	0.99 (0.48–2.05)	0.978
Tetracosanic acid 24:0	1.46 (0.81–2.62)	0.206	1.50 (0.84–2.67)	0.167	1.50 (0.74–3.03)	0.261
**MUFA**						
Palmitoleic acid 16:1*n − 7*	1.23 (0.77–1.96)	0.395	1.03 (0.63–1.66)	0.920	0.63 (0.33–1.20)	0.160
Cis vaccenic acid 18:1*n − 7*	1.54 (0.62–3.82)	0.355	1.84 (0.68–4.97)	0.231	1.43 (0.42–4.86)	0.569
Oleic acid 18:1*n − 9*	2.45 (1.23–4.88)	0.011	1.58 (0.75–3.32)	0.229	1.59 (0.57–4.44)	0.379
11 − eicosenoic acid 20:1*n − 9*	1.24 (0.84–1.84)	0.285	1.19 (0.78–1.81)	0.413	1.61 (0.87–2.98)	0.131
Nervonic acid 24:1*n − 9*	1.19 (0.70–2.02)	0.519	1.54 (0.91–2.60)	0.104	2.04 (1.05–3.96)	0.035
**n** − **6 PUFA**						
LA 18:2*n* − *6*^c^	0.72 (0.39–1.36)	0.314	1.23 (0.64–2.37)	0.527	2.32 (0.99–5.41)	0.052
DGLA 20:3*n* − *6*	0.78 (0.45–1.37)	0.388	0.76 (0.43–1.35)	0.350	1.18 (0.63–2.23)	0.603
AA 20:4*n* − *6*	0.74 (0.38–1.46)	0.388	0.79 (0.41–1.51)	0.418	1.40 (0.65–3.00)	0.388
Adrenic acid 22:4*n* − *6*	1.13 (0.60–2.13)	0.701	1.32 (0.76–2.30)	0.322	1.92 (0.96–3.87)	0.067
**n − 3 PUFA**						
ALA 18:3*n − 3*^d^	0.85 (0.57–1.28)	0.445	0.85 (0.57–1.25)	0.399	1.14 (0.72–1.80)	0.583
EPA 20:5*n − 3*	0.67 (0.49–0.91)	0.011	0.82 (0.61–1.10)	0.190	0.73 (0.51–1.05)	0.090
DPA 22:5*n − 3*	0.43 (0.25–0.74)	0.002	0.66 (0.41–1.06)	0.082	0.83 (0.43–1.62)	0.585
DHA 22:6*n − 3*	0.97 (0.58–1.65)	0.919	0.87 (0.52–1.46)	0.604	0.99 (0.57–1.73)	0.976
**Other**						
CLA 18:2*n − 7* ct/tc10,12	1.00 (0.77–1.30)	0.985	0.89 (0.70–1.14)	0.369	0.66 (0.47–0.94)	0.021
DMA16	0.98 (0.46–2.08)	0.954	1.13 (0.51–2.53)	0.758	1.28 (0.50–3.30)	0.612
DMA18	0.75 (0.32–1.78)	0.518	0.94 (0.35–2.53)	0.899	2.03 (0.59–6.98)	0.260
Ratio *n* − *6:n − 3* PUFA	1.01 (0.91–1.11)	0.908	1.09 (0.94–1.26)	0.260	1.20 (1.01–1.41)	0.036

^a^Conditional logistic regression analysis with centered log-ratio transformed variables (except for the ratio of sum n − 6 and sum n − 3) was adjusted for HLA genotype DR3/4, ancestry (PC1 and PC2), and weight z-score.

^b^Docosanoid acid (22:0) showed interaction with breastfeeding at 3 months of age (*p* = 0.027).

^c^LA showed interaction with any breastfeeding at 3 months of age (*p* = 0.038).

^d^ALA showed interaction with any breastfeeding at 3 months of age (*p* = 0.024).

**Table 3:** The difference between fatty acid status of breastfed and not breastfed children at the age of 3 and 6 months in TEDDY nested case–control study.

	At 3 months		At 6 months	
	Parameter estimate (SE)	*p* value^a^	Parameter estimate (SE)	*p* value^a^
**SFA**				
Myristic acid 14:0	− 0.11 (0.02)	< 0.0001	0.03 (0.02)	0.090
Pentadecanoic acid 15:0	0.21 (0.02)	< 0.0001	0.22 (0.02)	< 0.0001
Palmitic acid 16:0	− 0.25 (0.01)	< 0.0001	− 0.19 (0.01)	< 0.0001
Heptadecanoic acid 17:0	0.09 (0.02)	< 0.0001	0.14 (0.01)	< 0.0001
iso − heptadecanoic acid i17:0	0.91 (0.03)	< 0.0001	0.78 (0.03)	< 0.0001
Stearic acid 18:0	− 0.12 (0.01)	< 0.0001	− 0.09 (0.01)	< 0.0001
Eicosanoid acid 20:0	− 0.29 (0.02)	< 0.0001	− 0.24 (0.02)	< 0.0001
Docosanoic acid 22:0	− 0.18 (0.02)	< 0.0001	− 0.14 (0.02)	< 0.0001
Tetracosanic acid 24:0	− 0.22 (0.02)	< 0.0001	− 0.18 (0.02)	< 0.0001
**MUFA**				
Palmitoleic acid 16:1*n − 7*	0.34 (0.03)	< 0.0001	0.22 (0.02)	< 0.0001
Cis vaccenic acid 18:1*n − 7*	0.10 (0.01)	< 0.0001	0.06 (0.01)	< 0.0001
Oleic acid 18:1*n − 9*	− 0.25 (0.01)	< 0.0001	− 0.21 (0.01)	< 0.0001
11 − eicosenoic acid 20:1*n − 9*	− 0.32 (0.03)	< 0.0001	− 0.32 (0.02)	< 0.0001
Nervonic acid 24:1*n − 9*	− 0.34 (0.02)	< 0.0001	− 0.28 (0.02)	< 0.0001
**n** − **6 PUFA**				
LA 18:2*n* − *6*	− 0.34 (0.02)	< 0.0001	− 0.28 (0.01)	< 0.0001
DGLA 20:3*n* − *6*	− 0.04 (0.02)	0.042	− 0.09 (0.02)	< 0.0001
AA 20:4*n* − *6*	− 0.08 (0.02)	< 0.0001	− 0.11 (0.01)	< 0.0001
Adrenic acid 22:4*n* − *6*	− 0.26 (0.02)	< 0.0001	− 0.29 (0.02)	< 0.0001
**n − 3 PUFA**				
ALA 18:3*n − 3*	− 0.37 (0.03)	< 0.0001	− 0.34 (0.02)	< 0.0001
EPA 20:5*n − 3*	0.57 (0.05)	< 0.0001	0.48 (0.04)	< 0.0001
DPA 22:5*n − 3*	0.31 (0.02)	< 0.0001	0.32 (0.02)	< 0.0001
DHA 22:6*n − 3*	0.03 (0.02)	0.118	− 0.01 (0.02)	0.679
**Other**				
CLA 18:2*n − 7* ct/tc10,12	0.82 (0.04)	< 0.0001	0.73 (0.03)	< 0.0001
DMA16	− 0.16 (0.01)	< 0.0001	− 0.16 (0.01)	< 0.0001
DMA18	− 0.06 (0.01)	< 0.0001	− 0.06 (0.01)	< 0.0001
Ratio *n* − *6:n − 3* PUFA	− 1.36 (0.12)	< 0.0001	− 1.09 (0.07)	< 0.0001

^a^The difference between fatty acid status of breastfed and not breastfed children at the age of 3 and 6 month was tested by fitting a linear regression model for CLR transformed fatty acid (except for the ratio of sum n − 6 and sum n − 3), adjusted for case–control status.

Infants’ fatty acid status at the age of 3 and 6 months was not associated with the risk of multiple islet autoimmunity (Supplementary information Table 2), but some associations with IAA first and GADA first outcomes were observed. DPA (22:5*n* − *3*) at 3 months showed a protective association with IAA first autoimmunity, while a high ratio of *n* − *6:n* − *3* PUFA at 6 months was associated with a higher risk (Supplementary information Table 3). For GADA first, a protective association was observed for AA (20:4*n* − *6*) and adrenic acid (22:4*n* − *6*) at 6 months, while myristic acid (14:0) at 6 months was associated with a higher risk (Supplementary information Table 4).

### Erythrocyte fatty acid composition in children aged 1–6 years and the risk of islet autoimmunity

In childhood (1–6 years of age), CLA showed an inverse association with islet autoimmunity (Table 3). In contrast, higher stearic (18:0) and nervonic (24:1*n* − *9*) acids and a high ratio of *n* − *6:n* − *3* PUFA were associated with an increased risk of islet autoimmunity. Furthermore, stearic acid (18:0), cis vaccenic acid (18:1*n* − *7*), and dimethylacetal form of 18:0 (DMA18) were associated with a higher risk of multiple islet autoimmunity in childhood (Supplementary information Table 2). A high ratio of *n* − *6:n* − *3* PUFA was associated with an increased risk of IAA first (Supplementary information Table 3), while there were no associations for GADA first (Supplementary information Table 4).

None of the false discovery rate adjusted *p* values for the associations between fatty acids and the risk of islet autoimmunity, multiple islet autoimmunity, IAA first or GADA first were statistically significant.

## Discussion

Our study showed some associations between erythrocyte fatty acid composition and the risk of islet autoimmunity. EPA (20:5*n* − *3*) and DPA (22:5*n* − *3*) in early infancy were associated with a lower risk of islet autoimmunity. These fatty acids originate from the diet, but are synthesized endogenously from ALA (18:3*n* − *3*) also. ALA itself, as well as LA (18:2*n* − *6*) in early infancy, showed protective associations with islet autoimmunity in non-breastfed infants. The even-chain SFAs palmitic (16:0) and stearic acid (18:0) and MUFAs oleic (18:1*n* − *9*) and nervonic acid (24:1*n* − *9*) in infancy or childhood, reflecting mostly endogenous biosynthesis in the liver, were associated with an increased risk. On the other hand, CLA (18:2*n* − *7*) in childhood, obtained from dairy or synthesized endogenously, was associated with a lower risk. The observed associations were, however, not consistent across age. Furthermore, associations between fatty acids and islet autoimmunity differed by the type of outcome (islet autoimmunity, multiple, IAA first, and GADA first).

The results support the view that long-chain *n* − *3* PUFAs are protective, especially at an early age. They may affect the activation and development of the immune system in infancy, the maturation of the gut such as microbiota, permeability, and barrier function as well as inflammatory responses, with long-term consequences^1^. Our results are in line with some animal studies^28^ as well as two prospective studies^5–7^ although different *n* − *3* fatty acids (ALA, EPA, DPA, DHA) were associated with reduced risk in the different studies. This may be explained by differences in exposure measurements, outcomes and supplementation policies. The fact that our study indicates a protective role for EPA and DPA, and results from the DAISY study for DPA^6^, raises the question whether infants at risk of type 1 diabetes might benefit from supplementation with EPA and DPA also, not just DHA. The question is justified because EPA and DPA are precursors of different lipid mediators compared to DHA^29,30^. Altogether, the results indicate a possibility for preventive interventions by modification of fatty acid composition of early diets (e.g. infant formulas or breastmilk through changes in maternal diet). However, in the TEDDY study maternal intake of n − 3 fatty acid supplementation during pregnancy was not associated with the risk of islet autoimmunity in the offspring^31^. The ratio of *n* − *6:n* − *3* PUFA showed a positive association with islet autoimmunity and IAA first outcome in children 1–6 years of age suggesting that *n* − *3* PUFA may be protective among older children also. Furthermore, the results suggest that *n* − *3* PUFA may protect particularly against the development of primary insulin autoimmunity, which is in line with earlier findings^7^.

An important finding in this study was that the major even-chain SFAs [palmitic (16:0), stearic (18:0)], and MUFAs [oleic (18:1*n* − *9*) and nervonic (24:1*n* − *9*) acids], were associated with an increased risk of islet autoimmunity. Furthermore, for the multiple islet autoimmunity endpoint, stearic (18:0) and cis vaccenic acid (18:1*n* − *7*) showed increased risk. The above-mentioned fatty acids are mainly produced endogenously in the liver from shorter-chain fatty acids, as well as by de novo lipogenesis^32^. The increase in SFA and MUFA levels may reflect changes taking place in fatty acid metabolism, before islet autoimmunity. Interestingly, similar associations have been observed for type 2 diabetes in large prospective cohorts^32,33^ possibly reflecting some of the pathogenic disturbances caused by a failure in insulin secretion and signaling^24^. Even-chain SFAs could also have detrimental effects per se, e.g. palmitic acid (16:0) has been associated with activation of inflammatory cytokines and lipotoxicity in pancreatic beta cells^34^.

Breastfeeding status affected erythrocyte fatty acid composition in infants in the current study, which is in line with previous findings for serum fatty acids^7^. This is probably explained by differences in fatty acid content of breast milk and infant formula, but may also be caused by some other differences between the breastfed and formula-fed infants. Interestingly, breastfeeding in early infancy modified the association between ALA and LA status and the risk of islet autoimmunity. Higher ALA and LA status showed an inverse association in non-breastfed infants, while no association was seen in breastfed infants. The results indicate that an adequate intake of these essential fatty acids is even more important for infants not receiving any breast milk, and emphasize importance of fatty acid composition of infant formulas, the main source of the essential fatty acids in non-breastfed infants.

In our study, CLA (18:2*n* − *7*) was associated with a lower risk of islet autoimmunity in children aged 1–6 years. The main dietary source of CLA is dairy products, although it is also derived from fish and meat and it is produced endogenously to some degree^35^. CLA has been shown to exhibit various anti-inflammatory^36^, antiobesogenic and type 2 antidiabetic properties^37^. However, the protective association observed in our study may also be a consequence of increased *n* − *3* PUFA levels. CLA supplementation has been shown to increase plasma levels of EPA, for instance^38,39^. Our finding does not support the earlier prospective observation of positive associations between serum CLA and some dairy biomarkers and the risk of advanced islet autoimmunity^9^.

Strengths of the study include a nested case–control design within a large-scale birth cohort, a high number of islet autoimmunity cases, as well as prospectively collected data. Furthermore, we used fatty acid biomarkers, which reflect long-term dietary intake, biosynthesis, and metabolism. In addition, we analyzed a relatively large number of medium to long chain-length fatty acids from several biosynthetic pathways. We adjusted the results with weight because it is associated with both type 1 diabetes development^40,41^ and status of some of the fatty acids. The effect of weight adjustment was, however, relatively small. It can be considered a limitation that our study design does not allow us to draw causal inferences about the observed associations between erythrocyte fatty acid levels and the risk of islet autoimmunity. Further, we did not analyze maternal or child dietary intake of fatty acids. However, this will be done in future research. Also, our study population was selected on the basis of HLA-conferred risk of type 1 diabetes, which limits its generalizability to the whole population.

The current results confirm earlier prospective findings that long-chain *n* − *3* PUFA may protect from islet autoimmunity indicating possibility for early dietary intervention in terms of prevention. In addition, changes in the metabolism or intake of other fatty acids, such as even-chain SFAs and MUFAs, and CLA, may precede islet autoimmunity. Further studies are warranted to elucidate the role of individual fatty acids and fatty acid metabolism in type 1 diabetes etiology.

## Methods

### TEDDY cohort

**Figure 1:**
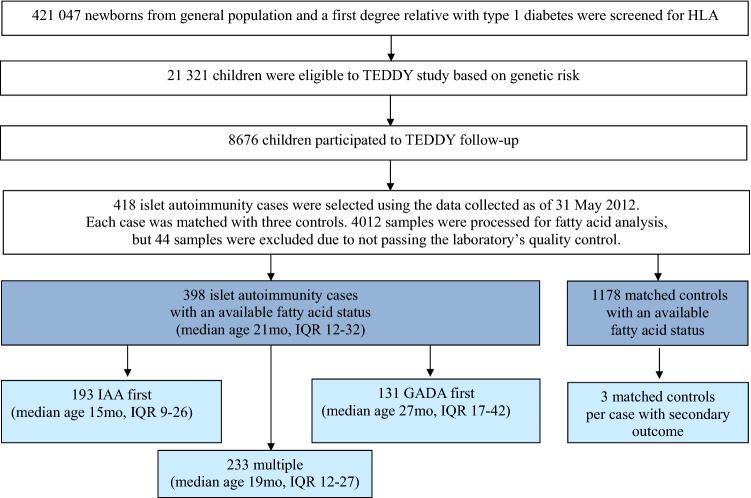
Flow chart of the study participants.

The current study was carried out in a nested case–control design within the international prospective the Environmental Determinants of Diabetes in the Young (TEDDY) birth cohort of children with increased genetic risk for type 1 diabetes. The study population was recruited between September 2004 and February 2010 in six clinical sites from the U.S. (Colorado, Georgia and Washington), Finland, Sweden, and Germany. The criteria for increased genetic risk were defined by HLA-associated risk genotypes separately for children from general population and children having a first degree relative with type 1 diabetes. In the general population, HLA-associated risk genotypes were DR3/4, DR3/3, DR4/4 and DR4/8^42^. Additional eligible genotypes were DR4/1, DR4/13, DR4/9, and DR3/9 in infants with first degree relative with type 1 diabetes. Of the screened 421 047 newborns, 21 321 were eligible based on the genetic risk and, of them 8676 participated to the follow up before age of 4 months (Fig. 1). Children are followed until the age of 15 years or type 1 diabetes diagnose at 3–6 months intervals. Autoantibodies for insulin autoantibodies (IAA), glutamate decarboxylase (GADA) and islet antigen 2 (IA-2A) were measured. Islet autoimmunity was defined as being persistent confirmed positive for at least one autoantibody out of the three measured. Written informed consent was obtained for all children from a parent and/or legal guardian. All methods were carried out in accordance with relevant guidelines and regulations. The TEDDY study was approved by the following ethical institutional review boards: the Colorado Multiple Institutional Review Board, the Hospital District of Southwest Finland Committee on Ethics, the University of Florida Health Center Institutional Review Board, the Augusta University Institutional Review Board (Georgia), the Ethik-Kommission der Bayerischen Landesarztekammer (Germany), the University of Pittsburgh Institutional Review Board, the Lund University Committee for Continuing Ethical Review (Sweden), the Western Institutional Review Board (Washington), and the University of South Florida Institutional Review Board. The study is also monitored by an External Evaluation Committee formed by the U.S. National Institutes of Health.

### A nested case–control design and outcomes

Children’s erythrocytes’ fatty acid composition was analyzed in a nested case–control design as described previously^43^. Matching factors were the clinical site, sex and family history of type 1 diabetes (first degree relative vs. not). A control was defined as a participant who had not developed persistent islet autoimmunity by the time when the corresponding matched case developed it, within ± 45 days of the event time. The nested case–control set was based on the data collected as of 31 May 2012. The study included 398 persistent islet autoimmunity cases with an available fatty acid status (385 cases with three controls; 10 cases with two controls; 3 cases with one control) (Fig. 1). In islet autoimmunity cases, median age of seroconversion was 21 months (interquartile range 12–32 months). Multiple islet autoimmunity (repeated positivity for at least two autoantibodies), primary positivity for IAA alone (IAA first), and GADA alone (GADA first) were analyzed as secondary outcomes. From 398 islet autoimmunity cases 233 had multiple islet autoimmunity, 193 had IAA first and 131 had GADA first outcomes. For multiple islet autoimmunity median age was 19 months (interquartile range 12–27), for IAA first 15 months (9–26 months) and for GADA first 27 months (17–42 months).

### Erythrocyte sample collection, processing and measurement of fatty acids

Blood samples were obtained from the children by venipuncture at the age of 3 and 6 months and 1, 2, 3, 4, 5, and 6 years at clinic visits. For the participants living far away from their nearest TEDDY clinic, a family periatrician collected the blood samples, which were sent to the TEDDY clinic within 24 h for processing (long distance protocol). All samples were aliquoted into dedicated, barcoded, and color-coded cryovials. To the blood sample used for fatty acid analysis, 2-propanol with 50 mg/L of butylated hydroxytoluene were added. The samples were then shipped frozen to the TEDDY Repository and immediately stored at − 80 °C. Collection and processing of samples are previously described in more detail^44^.

Fatty acids were analysed from erythrocytes by a gas chromatographic method modified from previously published methods^45,46^. Erythrocyte fatty acid composition was analysed using an Agilent 6890 gas chromatograph (Hewlett Packard, Palo Alto, CA, USA) with a split injector and hydrogen as the carrier gas. We employed a capillary column Omegawax 320 (length: 30 m, I.D.: 0.32 mm, phase layer: 0.25 µm; Supelco, Bellefonte, PA, USA). The percentage composition of fatty acid methyl esters was normalized to 100% in each sample. Samples of the cases and their controls at each age point were processed in the same batch to minimize potential batch effects. The laboratory was blinded regarding the case–control status of the samples. Total 4012 samples were processed for the islet autoimmunity analysis, but 44 samples were excluded due to not passing the laboratory’s quality control. The median number of analyzed samples per child was 3 (min = 1, max = 7). We determined altogether 25 different fatty acids.

### Dietary data

We collected information about breastfeeding duration, which was asked at the 3 and 6 months clinic visits. Parents or primary caretaker recorded the infant feeding information in a notebook that was given at the first clinical visit at 3 months. Clinical staff checked the booklet together with the primary caretaker at every clinical visit and entered the dietary information into the TEDDY database. The definition of any breastfeeding included breastfeeding, even in small amounts, and in combination with other foods. In the statistical analyses we used two categories for any breastfeeding: breastfed/not breastfed at cross-sectional time point either 3 or 6 months of age.

### Genetic measurements

Children in the study cohort were genotyped for the major type 1 diabetes associated class II haplotypes as well as for single-nucleotide polymorphisms (SNPs) defining type 1 diabetes risk outside HLA region^47^. Ancestry was estimated based on the principal components analysis (PCA)^48^ from the ImmunoChip data using the entire cohort. EIGENSTRAT software was used after selecting one subject per family. Two largest principal components were used in this study for defining population stratification.

### Statistical analysis

Fatty acid status for each child was generated as a percentage of the total 25 fatty acids. Since the sum is restricted to 100, the fatty acid status carries only a relative information, which may produce spurious findings without data normalization. Thus, we used the centered log-ratio (CLR) transformed fatty acid status for statistical comparisons, except for the ratio of sum *n* − *6* and sum *n* − *3* PUFA. Sum of *n* − *6* PUFA was obtained by summing up LA, dihomogammalinolenic acid (DGLA), arachidonic acid (AA) and adrenic acid. Sum of *n* − *3* PUFA was the sum of ALA, EPA, DPA and DHA. As the change after 1 year old was ignorable, we analyzed fatty acid status at early age (3 and 6 months), along with the average status from 1 to 6 years old. Conditional logistic regression examined the association between islet autoimmunity and fatty acid status after adjusting for HLA genotype, ancestry and weight z-score at the age corresponding to fatty acid status. The average weight from 1 to 6 years old was adjusted for the average status from 1 to 6 years old. Weight z score was obtained from Centers for Disease Control and Prevention standardized growth charts. Interaction between fatty acid status at early age and whether any breastfeeding took place at the corresponding age on the risk of islet autoimmunity was examined by testing an interaction term in the conditional logistic regression model. One unit change in a CLR transformed fatty acid status corresponds to the fatty acid status in percentage times 1.83. Association between fatty acid status at early age and the corresponding breastfeeding status was assessed using a linear regression model adjusted for the case–control status. Two-sided *p* values are reported. Statistical significance was determined when the *p* value was < 0.05. All statistical analyses were performed using SAS version 9.4 (SAS Institute Inc., Cary, NC).

Since 26 defined fatty acids were analyzed for each outcome, false discovery rate adjusted *p* values were calculated for multiple testing correction^49^.

## Supplementary Information

Supplementary Tables.

## Data Availability

The datasets generated and analyzed during the current study will be made available in the NIDDK Central Repository at https://www.niddkrepository.org/studies/teddy.

## Abbreviations

ALA: Alphalinolenic acid
CLA: Conjugated linoleic acid
DPA: Docosapentaenoic acid
DHA: Docosahexaenoic acid
DMA: Dimethylacetal
EPA: Eicosapentaenoic acid
GADA: Glutamic acid decarboxylase
HLA: Human leukocyte antigen
IAA: Insulin autoantibody
IA-2A: Insulinoma-associated antigen-2
LA: Linoleic acid
MUFA: Monounsaturated fatty acid
PUFA: Polyunsaturated fatty acid
SFA: Saturated fatty acid
SNP: Single nucleotide polymorphism
TEDDY: The Environmental Determinants of Diabetes in the Young Study
