# Supplementary material for: Children’s erythrocyte fatty acids are associated with the risk of islet autoimmunity
Source: Sci Rep. 2021 Feb 11;11:3627. doi: 10.1038/s41598-021-82200-9 (PMC7878879; doi:10.1038/s41598-021-82200-9)
Supplement: Supplementary file 1 — Supplementary Tables. [file 41598_2021_82200_MOESM1_ESM.docx]

**Supplementary information**

**Children’s erythrocyte fatty acids are associated with the risk of islet autoimmunity**

Sari Niinistö , Iris Erlund, Hye-Seung Lee, Ulla Uusitalo , Irma Salminen, Carin Andrén Aronsson, Hemang Parikh, Xiang Liu, Sandra Hummel, Jorma Toppari, Jin-Xiong She, Åke Lernmark, Annette G. Ziegler, Marian Rewers, Beena Akolkar, Jeffrey P. Krischer, David Galas, Siba Das, Nikita Sakhanenko, Stephen S. Rich, William Hagopian, Jill M. Norris, Suvi M. Virtanen, the TEDDY Study Group

Supplementary information Table 1. Erythrocyte fatty acid status in children in TEDDY nested case-control study

Supplementary information Table 2. The risk of multiple islet autoimmunity associated with erythrocyte fatty acid status in TEDDY nested case-control study.

Supplementary information Table 3. The risk of IAA first autoimmunity associated with erythrocyte fatty acid status in TEDDY nested case-control study.

Supplementary information Table 4. The risk of GADA first autoimmunity associated with erythrocyte fatty acid status in TEDDY nested case-control study.

**Supplementary information Table 1.** Erythrocyte fatty acid status in children in TEDDY nested case-control study.

|  | 3 months | | 6 months | | Mean over 1-6 years | |
| --- | --- | --- | --- | --- | --- | --- |
| Relative mean percentage of total fatty acids in erythrocytes ± SD | Cases  n=292 | Controls  n=732 | Cases  n=295 | Controls  n=815 | Cases  n=286 | Controls  n=826 |
| *SFA* |  |  |  |  |  |  |
| Myristic acid 14:0 | 0.33 **±** 0.09 | 0.34 **±** 0.09 | 0.30 **±** 0.10 | 0.30 **±** 0.10 | 0.29 **±** 0.08 | 0.29 **±** 0.08 |
| Pentadecanoic acid 15:0 | 0.12 **±** 0.07 | 0.12 **±** 0.04 | 0.11 **±** 0.04 | 0.11 **±** 0.05 | 0.14 **±**0.04 | 0.15 **±** 0.05 |
| Palmitic acid 16:0 | 21.46 **±** 2.23 | 21.28 **±** 2.05 | 21.69 **±** 1.96 | 21.35 **±** 1.91 | 21.85 **±** 1.53 | 21.80 **±** 1.67 |
| Heptadecanoic acid 17:0 | 0.33 **±** 0.10 | 0.33 **±** 0.08 | 0.30 **±** 0.09 | 0.30 **±** 0.08 | 0.34 **±** 0.07 | 0.34 **±** 0.07 |
| iso-heptadecanoic acid i17:0 | 0.07 **±** 0.04 | 0.07 **±** 0.03 | 0.07 **±** 0.03 | 0.07 **±** 0.03 | 0.10 **±** 0.04 | 0.10 **±** 0.04 |
| Stearic acid 18:0 | 13.19 **±** 1.83 | 13.09 **±** 1.66 | 12.59 **±** 1.46 | 12.57 **±** 1.49 | 12.33 **±** 1.39 | 12.20 **±** 1.30 |
| Eicosanoid acid 20:0 | 0.35 **±** 0.10 | 0.35 **±** 0.08 | 0.33 **±** 0.09 | 0.33 **±** 0.08 | 0.27 **±** 0.05 | 0.27 **±** 0.05 |
| Docosanoic acid 22:0 | 0.81 **±** 0.25 | 0.81 **±** 0.22 | 0.83 **±** 0.24 | 0.82 **±** 0.22 | 0.84 **±** 0.21 | 0.85 **±** 0.20 |
| Tetracosanic acid 24:0 | 2.14 **±** 0.59 | 2.11 **±** 0.57 | 2.17 **±** 0.64 | 2.14 **±** 0.60 | 2.23 **±** 0.58 | 2.21 **±** 0.52 |
| *MUFA* |  |  |  |  |  |  |
| Palmitoleic acid 16:1*n-7* | 0.24 **±** 0.11 | 0.24 **±** 0.10 | 0.22 **±** 0.09 | 0.23 **±** 0.10 | 0.24 **±** 0.09 | 0.25 **±** 0.09 |
| Cis vaccenic acid 18:1*n-7* | 1.63 **±** 0.38 | 1.63 **±** 0.33 | 1.48 **±** 0.30 | 1.48 **±** 0.28 | 1.44 **±** 0.18 | 1.44 **±** 0.18 |
| Oleic acid 18:1*n-9* | 15.40 **±** 2.99 | 15.16 **±** 2.86 | 15.53 **±** 2.32 | 15.50 **±** 2.50 | 15.17 **±** 1.95 | 15.27 **±** 2.05 |
| 11-eicosenoic acid 20:1*n-9* | 0.41 **±** 0.28 | 0.41 **±** 0.25 | 0.37 **±** 0.19 | 0.37 **±** 0.19 | 0.37 ± 0.13 | 0.37 ± 0.14 |
| Nervonic acid 24:1*n-9* | 2.66 **±** 0.77 | 2.63 **±** 0.71 | 2.66 **±** 0.82 | 2.60 **±** 0.73 | 2.74 ± 0.71 | 2.68 ± 0.69 |
| *n-6 PUFA* |  |  |  |  |  |  |
| LA 18:2*n-6* | 8.63 **±** 1.59 | 8.74 **±** 1.52 | 9.75 **±** 1.63 | 9.76 **±** 1.63 | 10.43 ± 1.45 | 10.36 ± 1.46 |
| DGLA 20:3*n-6* | 1.39 **±** 0.36 | 1.41 **±** 0.34 | 1.17 **±** 0.30 | 1.20 **±** 0.31 | 1.31 ± 0.32 | 1.32 ± 0.31 |
| AA 20:4*n-6* | 12.38 **±** 2.67 | 12.59 **±** 2.63 | 12.16 **±** 2.38 | 12.37 **±** 2.37 | 12.19 ±2.02 | 12.25 ± 2.10 |
| Adrenic acid 22:4*n-6* | 3.11 **±** 0.74 | 3.09 **±** 0.67 | 2.93 **±** 0.67 | 2.91 **±** 0.70 | 3.19 ± 0.73 | 3.15 ± 0.73 |
| *n-3 PUFA* |  |  |  |  |  |  |
| ALA 18:3*n-3* | 0.09 **±** 0.04 | 0.09 **±** 0.03 | 0.11 **±** 0.04 | 0.11 **±** 0.04 | 0.14 ± 0.05 | 0.15 ± 0.05 |
| EPA 20:5*n-3* | 0.42 **±** 0.27 | 0.47 **±** 0.30 | 0.43 **±** 0.25 | 0.47 **±** 0.29 | 0.47 ± 0.26 | 0.51 ± 0.29 |
| DPA 22:5*n-3* | 1.61 **±** 0.50 | 1.71 **±** 0.49 | 1.67 **±** 0.53 | 1.75 **±** 0.54 | 1.86 ± 0.49 | 1.90 ± 0.50 |
| DHA 22:6*n-3* | 5.23 **±** 1.31 | 5.29 **±** 1.37 | 4.97 **±** 1.31 | 5.02 **±** 1.27 | 3.93 ± 0.99 | 3.99 ± 1.08 |
| *Other* |  |  |  |  |  |  |
| CLA 18:2*n-7* ct/tc10,12 | 0.08 **±** 0.05 | 0.08 **±** 0.04 | 0.07 **±** 0.04 | 0.07 **±** 0.04 | 0.08 ± 0.04 | 0.09 ± 0.04 |
| DMA16 | 2.94 **±** 0.49 | 2.95 **±** 0.47 | 3.06 **±** 0.45 | 3.07 **±** 0.46 | 2.88 ± 0.41 | 2.89 ± 0.39 |
| DMA18 | 4.95 **±** 0.75 | 5.01 **±** 0.70 | 5.03 **±** 0.66 | 5.09 **±** 0.69 | 5.17 ± 0.59 | 5.18 ± 0.60 |

**Supplementary information Table 2.** The risk of multiple islet autoimmunity associated with erythrocyte fatty acid status in TEDDY nested case-control study.

|  | Multiple islet autoimmunity, Cases n=233 | | | | | |
| --- | --- | --- | --- | --- | --- | --- |
|  | 3 months | | 6 months | | Mean over 1-6 years | |
| Relative proportion (%) of total fatty acids in erythrocyte membrane | OR (95% CI)^a^ | p-value | OR (95% CI)^a^ | p-value | OR (95% CI)^a^ | p-value |
| *SFA* |  |  |  |  |  |  |
| Myristic acid 14:0 | 0.53 (0.23-1.25) | 0.146 | 1.26 (0.64-2.47) | 0.512 | 0.52 (0.18-1.47) | 0.215 |
| Pentadecanoic acid 15:0 | 1.00 (0.49-2.03) | 0.988 | 1.31 (0.61-2.86) | 0.489 | 0.46 (0.19-1.11) | 0.085 |
| Palmitic acid 16:0 | 0.90 (0.26-3.14) | 0.864 | 2.83 (0.78-10.25) | 0.113 | 3.49 (0.61-19.92) | 0.160 |
| Heptadecanoic acid 17:0 | 1.70 (0.52-5.57) | 0.383 | 1.64 (0.64-4.47) | 0.287 | 1.52 (0.36-6.47) | 0.569 |
| iso-heptadecanoic acid i17:0 | 1.14 (0.81-1.61) | 0.455 | 0.97 (0.70-1.34) | 0.841 | 0.72 (0.43-1.20) | 0.209 |
| Stearic acid 18:0 | 1.30 (0.30-5.57) | 0.723 | 3.30 (0.73-15.05) | 0.123 | **6.16 (1.37-27.76)** | **0.018** |
| Eicosanoid acid 20:0 | 1.12 (0.57-2.20) | 0.740 | 1.09 (0.50-2.34) | 0.833 | 1.32 (0.49-3.54) | 0.583 |
| Docosanoic acid 22:0 | 0.83 (0.37-1.86) | 0.643 | 1.10 (0.48-2.50) | 0.830 | 1.08 (0.41-2.83) | 0.874 |
| Tetracosanic acid 24:0 | 1.09 (0.49-2.44) | 0.839 | 1.36 (0.63-2.91) | 0.432 | 1.71 (0.67-4.32) | 0.260 |
| *MUFA* |  |  |  |  |  |  |
| Palmitoleic acid 16:1*n-7* | 1.06 (0.58-1.96) | 0.844 | 1.01 (0.55-1.86) | 0.967 | 0.77 (0.34-1.75) | 0.527 |
| Cis vaccenic acid 18:1*n-7* | 2.94 (0.86-10.03) | 0.086 | 3.23 (0.92-11.33) | 0.068 | **8.01 (1.47-43.55)** | **0.016** |
| Oleic acid 18:1*n-9* | 1.21 (0.50-2.90) | 0.676 | 0.93 (0.35-2.45) | 0.878 | 2.14 (0.59-7.73) | 0.244 |
| 11-eicosenoic acid 20:1*n-9* | 1.11 (0.67-1.84) | 0.689 | 1.00 (0.59-1.69) | 0.984 | 1.71 (0.78-3.74) | 0.182 |
| Nervonic acid 24:1*n-9* | 0.59 (0.29-1.23) | 0.159 | 1.10 (0.54-2.21) | 0.801 | 1.99 (0.84-4.74) | 0.120 |
| *n-6 PUFA* |  |  |  |  |  |  |
| LA 18:2*n-6* | 0.72 (0.31-1.68) | 0.452 | 1.19 (0.51-2.80) | 0.693 | 2.23 (0.71-7.04) | 0.172 |
| DGLA 20:3*n-6* | 1.07 (0.49-2.30) | 0.873 | 0.67 (0.31-1.44) | 0.305 | 1.09 (0.48-2.51) | 0.832 |
| AA 20:4*n-6* | 1.20 (0.46-3.14) | 0.708 | 0.89 (0.37-2.13) | 0.794 | 1.31 (0.45-3.80) | 0.617 |
| Adrenic acid 22:4*n-6* | 1.50 (0.61-3.68) | 0.381 | 1.26 (0.63-2.53) | 0.507 | 2.09 (0.75-5.84) | 0.159 |
| *n-3 PUFA* |  |  |  |  |  |  |
| ALA 18:3*n-3* | 0.70 (0.40-1.21) | 0.197 | 0.67 (0.39-1.17) | 0.162 | 0.74 (0.39-1.43) | 0.375 |
| EPA 20:5*n-3* | 0.77 (0.51-1.16) | 0.215 | 0.80 (0.54-1.18) | 0.259 | 0.65 (0.40-1.06) | 0.083 |
| DPA 22:5*n-3* | 0.63 (0.29-1.37) | 0. 243 | 0.76 (0.40-1.45) | 0.401 | 0.79 (0.33-1.90) | 0.602 |
| DHA 22:6*n-3* | 1.33 (0.59-2.97) | 0.495 | 1.16 (0.59-2.29) | 0.663 | 1.11 (0.54-2.30) | 0.783 |
| *Other* |  |  |  |  |  |  |
| CLA 18:2*n-7* ct/tc10,12 | 1.13 (0.79-1.61) | 0.520 | 0.93 (0.66-1.29) | 0.649 | 0.61 (0.37-1.01) | 0.052 |
| DMA16 | 1.91 (0.60-6.11) | 0.274 | 0.97 (0.34-2.77) | 0.955 | 1.48 (0.42-5.24) | 0.543 |
| DMA18 | 1.20 (0.32-4.47) | 0.788 | 0.87 (0.26-2.91) | 0.818 | **6.41 (1.15-35.74)** | **0.034** |
| Ratio *n-6:n-3* PUFA | 0.93 (0.74-1.16) | 0.498 | 0.99 (0.82-1.19) | 0.927 | 1.15 (0.92-1.43) | 0.210 |

^a^Conditional logistic regression analysis with centered log-ratio transformed variables (except for the ratio of sum n-6 and sum n-3) was adjusted for HLA genotype DR3/4, ancestry (PC1 and PC2) and weight z-score.

**Supplementary information Table 3.** The risk of IAA first autoimmunity associated with erythrocyte fatty acid status in TEDDY nested case-control study.

|  | IAA first autoimmunity, Cases n=193 | | | | | |
| --- | --- | --- | --- | --- | --- | --- |
|  | 3 months | | 6 months | | Mean over 1-6 years | |
| Relative proportion (%) of total fatty acids in erythrocyte membrane | OR (95% CI)^a^ | p-value | OR (95% CI)^a^ | p-value | OR (95% CI)^a^ | p-value |
| *SFA* |  |  |  |  |  |  |
| Myristic acid 14:0 | 0.69 (0.28-1.69) | 0.415 | 0.71 (0.33-1.52) | 0.382 | 0.95 (0.33-2.72) | 0.922 |
| Pentadecanoic acid 15:0 | 1.08 (0.48-2.41) | 0.855 | 0.65 (0.30-1.39) | 0.266 | 0.57 (0.21-1.57) | 0.277 |
| Palmitic acid 16:0 | 1.17 (0.30-4.60) | 0.818 | 2.20 (0.53-9.16) | 0.279 | 3.89 (0.53-28.72) | 0.183 |
| Heptadecanoic acid 17:0 | 1.24 (0.39-3.95) | 0.714 | 0.85 (0.30-2.41) | 0.764 | 0.97 (0.21-4.55) | 0.966 |
| iso-heptadecanoic acid i17:0 | 1.00 (0.69-1.46) | 0.991 | 0.87 (0.61-1.22) | 0.415 | 0.74 (0.41-1.33) | 0.312 |
| Stearic acid 18:0 | 2.28 (0.50-10.43) | 0.290 | 1.04 (0.21-5.18) | 0.964 | 4.35 (0.82-22.99) | 0.084 |
| Eicosanoid acid 20:0 | 1.45 (0.67-3.12) | 0.342 | 0.81 (0.35-1.86) | 0.617 | 1.43 (0.47-4.34) | 0.526 |
| Docosanoic acid 22:0 | 1.18 (0.56-2.48) | 0.658 | 0.92 (0.40-2.14) | 0.853 | 0.97 (0.32-2.90) | 0.952 |
| Tetracosanic acid 24:0 | 1.59 (0.69-3.65) | 0.273 | 1.22 (0.55-2.69) | 0.621 | 1.37 (0.47-4.00) | 0.568 |
| *MUFA* |  |  |  |  |  |  |
| Palmitoleic acid 16:1*n-7* | 1.20-0.65-2.22 | 0.557 | 0.95 (0.50-1.82) | 0.883 | 0.83 (0.32-2.16) | 0.705 |
| Cis vaccenic acid 18:1*n-7* | 1.85 (0.48-7.10) | 0.368 | 1.17 (0.29-4.79) | 0.828 | 4.05 (0.56-29.24) | 0.166 |
| Oleic acid 18:1*n-9* | 1.89 (0.71-5.03) | 0.201 | 1.71 (0.57-5.09) | 0.339 | 3.62 (0.80-16.38) | 0.095 |
| 11-eicosenoic acid 20:1*n-9* | 1.20 (0.71-2.02) | 0.503 | 1.21 (0.65-2.25) | 0.550 | 1.43 (0.60-3.44) | 0.423 |
| Nervonic acid 24:1*n-9* | 1.27 (0.60-2.68) | 0.532 | 1.34 (0.65-2.73) | 0.426 | 1.83 (0.69-4.82) | 0.225 |
| *n-6 PUFA* |  |  |  |  |  |  |
| LA 18:2*n-6* | 0.69 (0.28-1.69) | 0.411 | 2.01 (0.83-4.88) | 0.121 | 3.02 (0.79-11.57) | 0.107 |
| DGLA 20:3*n-6* | 1.15 (0.51-2.60) | 0.743 | 1.02 (0.46-2.27) | 0.962 | 1.16 (0.43-3.17) | 0.767 |
| AA 20:4*n-6* | 1.00 (0.39-2.59) | 0.999 | 1.29 (0.51-3.26) | 0.593 | 1.28 (0.41-3.99) | 0.669 |
| Adrenic acid 22:4*n-6* | 1.23 (0.53-2.88) | 0.633 | 2.09 (0.94-4.63) | 0.071 | 2.83 (1.00-8.03) | 0.051 |
| *n-3 PUFA* |  |  |  |  |  |  |
| ALA 18:3*n-3* | 0.93 (0.51-1.68) | 0.797 | 1.04 (0.59-1.83) | 0.889 | 1.09 (0.60-1.98) | 0.783 |
| EPA 20:5*n-3* | 0.71 (0.47-1.07) | 0.105 | 0.89 (0.58-1.37) | 0.604 | 0.60 (0.35-1.01) | 0.056 |
| DPA 22:5*n-3* | **0.45 (0.22-0.93)** | **0.031** | 1.00 (0.50-1.98) | 0.990 | 0.76 (0.30-1.94) | 0.563 |
| DHA 22:6*n-3* | 1.08 (0.55-2.14) | 0.822 | 0.63 (0.30-1.34) | 0.230 | 0.64 (0.28-1.48) | 0.293 |
| *Other* |  |  |  |  |  |  |
| CLA 18:2*n-7* ct/tc10,12 | 0.98 (0.67-1.44) | 0.925 | 0.83 (0.58-1.21) | 0.333 | 0.63 (0.38-1.05) | 0.074 |
| DMA16 | 0.73 (0.29-1.86) | 0.513 | 2.54 (0.81-7.97) | 0.110 | 1.75 (0.42-7.29) | 0.443 |
| DMA18 | 0.62 (0.21-1.79) | 0.376 | 1.96 (0.50-7.59) | 0.333 | 3.19 (0.48-21.10) | 0.230 |
| Ratio *n-6:n-3* PUFA | 1.00 (0.90-1.12) | 0.967 | **1.24 (1.01-1.53)** | **0.038** | **1.41 (1.09-1.84)** | **0.010** |

^a^Conditional logistic regression analysis with centered log-ratio transformed variables (except for the ratio of sum n-6 and sum n-3) was adjusted for HLA genotype DR3/4, ancestry (PC1 and PC2) and weight z-score.

**Supplementary information Table 4.** The risk of GADA first autoimmunity associated with erythrocyte fatty acid status in TEDDY nested case-control study.

|  | GADA first autoimmunity, Cases n=131 | | | | | |
| --- | --- | --- | --- | --- | --- | --- |
|  | 3 months | | 6 months | | Mean over 1-6 years | |
| Relative proportion (%) of total fatty acids in erythrocyte membrane | OR (95% CI)^a^ | p-value | OR (95% CI)^a^ | p-value | OR (95% CI)^a^ | p-value |
| *SFA* |  |  |  |  |  |  |
| Myristic acid 14:0 | 1.20 (0.42-3.45) | 0.740 | **2.81 (1.09-7.19)** | **0.032** | 1.52 (0.41-5.66) | 0.530 |
| Pentadecanoic acid 15:0 | 1.92 (0.65-5.64) | 0.239 | 1.80 (0.55-5.82) | 0.329 | 0.79 (0.24-2.61) | 0.703 |
| Palmitic acid 16:0 | 3.67 (0.63-21.50) | 0.149 | 2.98 (0.60-14.80) | 0.183 | 1.02 (0.09-10.99) | 0.990 |
| Heptadecanoic acid 17:0 | 4.20 (0.73-24.10) | 0.107 | 3.35 (0.65-17.20) | 0.148 | 0.82 (0.14-4.66) | 0.819 |
| iso-heptadecanoic acid i17:0 | 1.07 (0.66-1.74) | 0.798 | 0.98 (0.64-1.51) | 0.934 | 0.74 (0.36-1.54) | 0.425 |
| Stearic acid 18:0 | 1.56 (0.19-12.46) | 0.678 | 2.39 (0.33-17.52) | 0.392 | 4.27 (0.58-31.25) | 0.153 |
| Eicosanoid acid 20:0 | 0.84 (0.25-2.75) | 0.767 | 1.73 (0.59-5.06) | 0.316 | 1.49 (0.40-5.57) | 0.555 |
| Docosanoic acid 22:0 | 0.95 (0.30-3.01) | 0.934 | 1.87 (0.57-6.12) | 0.304 | 1.46 (0.42-5.07) | 0.555 |
| Tetracosanic acid 24:0 | 1.18 (0.40-3.46) | 0.765 | 1.65 (0.56-4.88) | 0.369 | 1.81 (0.56-5.91) | 0.323 |
| *MUFA* |  |  |  |  |  |  |
| Palmitoleic acid 16:1*n-7* | 1.27 (0.48-3.37) | 0.634 | 1.31 (0.52-3.30) | 0.561 | 0.43 (0.14-1.37) | 0.153 |
| Cis vaccenic acid 18:1*n-7* | 0.92 (0.21-4.01) | 0.914 | 1.30 (0.20-8.30) | 0.783 | 0.38 (0.08-1.94) | 0.245 |
| Oleic acid 18:1*n-9* | 3.03 (0.74-12.39) | 0.123 | 1.18 (0.32-4.43) | 0.801 | 0.48 (0.07-3.31) | 0.456 |
| 11-eicosenoic acid 20:1*n-9* | 1.22 (0.55-2.71) | 0.627 | 0.89 (0.42-1.86) | 0.750 | 1.16 (0.32-4.15) | 0.825 |
| Nervonic acid 24:1*n-9* | 1.29 (0.49-3.40) | 0.608 | 1.88 (0.71-4.99) | 0.203 | 2.49 (0.77-8.04) | 0.128 |
| *n-6 PUFA* |  |  |  |  |  |  |
| LA 18:2*n-6* | 0.68 (0.21-2.22) | 0.519 | 0.46 (0.13-1.60) | 0.219 | 1.26 (0.32-5.02) | 0.742 |
| DGLA 20:3*n-6* | 0.60 (0.20-1.77) | 0.354 | 0.60 (0.22-1.67) | 0.328 | 1.12 (0.38-3.20) | 0.852 |
| AA 20:4*n-6* | 0.37 (0.10-1.41) | 0.144 | **0.26 (0.08-0.90)** | **0.033** | 0.99 (0.28-3.45) | 0.982 |
| Adrenic acid 22:4*n-6* | 0.33 (0.09-1.29) | 0.111 | **0.29 (0.10-0.91)** | **0.033** | 0.97 (0.29-3.22) | 0.960 |
| *n-3 PUFA* |  |  |  |  |  |  |
| ALA 18:3*n-3* | 0.94 (0.45-1.98) | 0.872 | 0.80 (0.42-1.52) | 0.488 | 1.09 (0.46-2.57) | 0.843 |
| EPA 20:5*n-3* | 0.95 (0.50-1.81) | 0.882 | 0.97 (0.57-1.64) | 0.905 | 0.96 (0.53-1.76) | 0.903 |
| DPA 22:5*n-3* | 0.52 (0.18-1.47) | 0.215 | 0.54 (0.24-1.23) | 0.143 | 1.05 (0.33-3.30) | 0.934 |
| DHA 22:6*n-3* | 0.97 (0.30-3.20) | 0.962 | 1.11 (0.45-2.73) | 0.818 | 1.04 (0.41-2.63) | 0.934 |
| *Other* |  |  |  |  |  |  |
| CLA 18:2*n-7* ct/tc10,12 | 0.96 (0.58-1.57) | 0.867 | 1.11 (0.71-1.73) | 0.662 | 0.92 (0.51-1.67) | 0.783 |
| DMA16 | 0.55 (0.10-2.88) | 0.475 | 0.25 (0.05-1.20) | 0.084 | 0.59 (0.12-2.91) | 0.517 |
| DMA18 | 0.40 (0.06-2.53) | 0.330 | 0.16 (0.02-1.18) | 0.072 | 0.57 (0.08-4.14) | 0.576 |
| Ratio *n-6:n-3* PUFA | 0.88 (0.62-1.25) | 0.480 | 0.84 (0.63-1.12) | 0.229 | 1.05 (0.79-1.41) | 0.722 |

^a^Conditional logistic regression analysis with centered log-ratio transformed variables (except for the ratio of sum n-6 and sum n-3) was adjusted for HLA genotype DR3/4, ancestry (PC1 and PC2) and weight z-score.
